# Knowledge, Information Needs and Risk Perception about HIV and Sexually Transmitted Diseases after an Education Intervention on Italian High School and University Students

**DOI:** 10.3390/ijerph18042069

**Published:** 2021-02-20

**Authors:** Antonella Zizza, Marcello Guido, Virginia Recchia, Pierfrancesco Grima, Federico Banchelli, Andrea Tinelli

**Affiliations:** 1 Institute of Clinical Physiology, National Research Council, 73100 Lecce, Italy; zizza@ifc.cnr.it (A.Z.); recchia@ifc.cnr.it (V.R.); 2 Laboratory of Hygiene, Department of Biological and Environmental Sciences and Technologies, University of Salento, 73100 Lecce, Italy; marcello.guido@unisalento.it; 3 Infectious Diseases Operative Unit, Vito Fazzi Hospital, 73100 Lecce, Italy; pierfrancescogrima@yahoo.it; 4 Department of Medical and Surgical Science, University of Modena and Reggio Emilia, 41125 Modena, Italy; federico.banchelli@unimore.it; 5 Department of Obstetrics and Gynecology, “Veris delli Ponti” Hospital, Scorrano, 73020 Lecce, Italy; 6 Division of Experimental Endoscopic Surgery, Imaging, Technology and Minimally Invasive Therapy, Vito Fazzi Hospital, 73100 Lecce, Italy

**Keywords:** sexually transmitted diseases, HIV, education, knowledge, risk, prevention and control, students, communication, surveys and questionnaires

## Abstract

Sexually transmitted diseases (STDs) among adolescents and young people represent a significant public health problem that generates a pressing requirement of effective evidence-based education to promote primary and secondary prevention. The objective of the study is to evaluate how knowledge, information needs, and risk perception about HIV and STDs can change after targeted education interventions for students. A total of 436 subjects aged 15–24 attending high school (134 biomedical and 96 non-biomedical fields) and university courses (104 scientific and 102 non-scientific disciplines) were enrolled to respond to a questionnaire before and after the intervention. An improvement in knowledge was found in all groups, with statistically significant knowledge score differences between the four groups in 60% of the items. More than 94% of the students consider it useful to promote information on these issues. Receiving this information generated awareness and safety in more than 85% of high-school students and 93% of University students. Students widely perceived a great risk being infected with HIV/STDs, although pregnancy was seen as a more hazardous consequence of unprotected sex. This study shows that educational interventions are effective in improving knowledge, apart from findings about key knowledge topics, information needs, and risk perception, which provide significant insights to design future targeted education programs.

## 1. Introduction

Sexually transmitted diseases (STDs) among adolescents and young people represent a huge problem, which requires effective interventions for primary and secondary prevention [1]. Although STDs mostly occur in developing countries, especially in the African region [2], the trend continues to increase even in industrialized countries [3,4]. Over 100 million new STDs, excluding HIV, occur every year among people under 25 years of age, and globally, more than half of subjects newly infected by HIV are young people aged 15–24 [5]. For women, the risk of infection in adolescence or early adulthood is even higher due to a greater anatomical susceptibility to these diseases [6]. The latest European data provided by the European Center for Disease prevention and Control show, every year, an increasing number of cases of the STDs recognized as curable [7].

The STDs affect predominantly adolescent and young people, probably for the following reasons: (a) a more frequent change of sexual partners, (b) a more frequent choice of sexual partners on the internet, (c) a poor information on how to prevent STDs, and (d) an excessive optimism regarding the availability/existence of effective drugs for HIV [8]. Earliness in the first sexual intercourse and the reluctance of young people to use condoms are additional causes of increasing number of STD cases [9,10].

In Italy, the cases of STDs increased, between 2000 and 2018, by 32.0% among women, by 27.0% among men, and almost three-fold, among men who have sex with men (MSM). Nevertheless, the 19.5% of all new Italian cases of STDs affect young people (15–24 years old) [11].

Furthermore, recent research has found that many Italian adolescents have their first sexual experience very early (15.6 ± 1.6 years) [12], have sexual intercourses with multiple partners, and are often unprotected [13].

These results highlight the need of ensuring education programs in this topic, although the association between education and STDs has shown conflicting evidence. In fact, in the early HIV epidemic, higher HIV infections were found among more educated people [14,15], while more recent studies indicated that education acted as a social vaccine against HIV risk [16,17]. Worldwide, numerous scientists assessed the level of knowledge about these infections among students of different ages and attending different types of schools and universities [6,12,18,19], while others evaluated the impact of STDs educational interventions [1,20,21,22,23,24]. An interesting theoretical framework employed in prevention programs regarding both STDs infections and other health-related risks is based on exploring “knowledge”, “information needs”, and “risk perception” [25,26,27,28,29,30,31,32].

The main objective of this investigation was to evaluate how knowledge, information needs, and risk perception about HIV and STDs can change after targeted education interventions for high school and university students with different education backgrounds, as well as to ascertain the main weak knowledge topics for each target group.

The final aim was to offer conceptual and practical contributions to design future education programs for primary and secondary prevention purposes, such as deterring risky behaviors or promoting the use of vaccines, and the early diagnosis.

## 2. Materials and Methods

### 2.1. Study Design and Population

A didactic intervention was designed and carried out from September 2018 to February 2019 for students attending high schools and universities in a city located in Southeast of Italy. A cohort of students of both sexes aged between 15 and 24 years, attending high schools in biomedical and financial management, as well as university courses in biotechnology, economics, cultural heritage, and archaeology, was invited to participate.

The questionnaire was given to all the students who agreed to participate, in order to collect data on their information needs, knowledge, and risk perception about HIV and STDs, before and after the educational intervention. In order to ensure anonymity, a numerical code was randomly assigned to each of the participants before filling out the questionnaire, and the same code was used for incoming and outgoing questionnaires.

Participating students were divided into four groups, based on their different degree and field of study. The project design foresaw 150 students per group. Two groups consisted of high school students in the biomedical and non-biomedical fields, Group 1 (G1) and Group 2 (G2), respectively, while the other two groups comprised students of scientific and non-scientific degree courses, Group 3 (G3) and Group 4 (G4), respectively. The sample size has been indirectly estimated on the basis of a chi-square test for comparing two equally-sized independent groups with respect to a binary outcome. The proportion of subjects with the outcome was assumed to be 60% in one group and 75% in the other, which yields an Odds Ratio equal to 2. To achieve 95% confidence level and 80% statistical power, 152 subjects per each group would be necessary.

### 2.2. Instrument

A self-administered ad-hoc questionnaire was designed on the basis of available scientific evidence regarding surveys on equivalent topics [33,34,35,36,37].

A group of experts in the fields of STDs, risk communication, and risk perception verified the content of the questionnaire and approved its administration, after some recommended adjustments. A pre-test of the questionnaire was performed on 10 students of 14–24 years, to evaluate the feasibility of the questionnaire for a large cohort of students. The interviewees were asked to comment on the questions and answers. The questions that were ambiguous or unclear by at least two respondents were rephrased. This allowed a few final tunings regarding the items’ wording.

The final version of the questionnaire consisted of a total of 35 items, included into four sections: demographic characteristics and socio-cultural context (Section A), information needs (Section B), knowledge about HIV and STDs (Section C), and risk perception (Section D) (Supplementary Materials).

Socio-demographic factors of students and their parents were evaluated for each group through 10 items included in Section A of the questionnaire. Data on age, gender, religion, relationship status, household living arrangement, employment status of students, education level, and occupation of parents were collected.

The information needs were evaluated through four items in Section B. Those questions explored a self-evaluation of the level of information about HIV and STDs and the utility to promote it, as well as the usefulness of receiving information on these issues and the most appropriate sources.

Section C was based on the topics included in the educational intervention; the 15 items focused mainly on transmission, prevention, diagnosis, and complications of HIV and major STDs. The knowledge level was measured using all those 15 items, and it was conveyed as the percentage of students—stratified into the four groups—who gave the correct answer for each question before and after the intervention. One point was assigned for each correct answer, and zero points for incorrect, unknown, or “not given” answer.

Finally, Section D included five items for assessing risk perception on a three-point Likert scale with “A lot”, “Moderately”, and “Not at all” as possible answers, and one item regarding the consequences of having unprotected sexual intercourse. The perceptions investigated relate to the concern of being infected with STDs and their danger, as well as the safety in the use of condoms and in the sexual life of peers.

### 2.3. Ethics Approval and Consent to Participate

The study was conducted in accordance with the Declaration of Helsinki and was approved by the Ethics Committee of the Local Health Unit (LHU) of Lecce (Report No. 19 dated 14 May 2018). Students over the age of 18 and parents of students under the age of 18 have given consent for participation in the study.

### 2.4. Statistical Analysis

Proportions, mean, minimum, and maximum were used for the descriptive analyses. For continuous variables, comparison between two groups was performed by two-tailed unpaired Student t test, whereas one-way ANOVA was used to compare the difference between more than two groups. For categorical variables, the chi-square test and Fisher’s exact test were used to examine the differences between groups.

Within groups, differences in knowledge were assessed by using the Wilcoxon signed-rank test for ordinal variables or the paired McNemar test for nominal variables.

The comparison in knowledge improvement between groups (G2 vs. G1 and G4 vs. G3) was assessed by using the Odds Ratio (OR) with 95% confidence interval (95% CI). The OR was estimated from a logistic regression model that adjusts for baseline knowledge.

The statistical analysis was performed with SPSS Statistic software (version 24.0 2016) (IBM Corp., Armonk, NY, USA) and R (version 3.6.3) (The R Foundation for statistical computing, Wien, Austria) software, and *p*-value < 0.05 was assumed as the level of statistical significance.

## 3. Results

### 3.1. Demographic and Socio-Cultural Characteristics

In total, 528 incoming and 467 outgoing questionnaires were administered. Only 436 questionnaires correctly filled were considered valid for the study, since incomplete questionnaires and those with discrepancies between entry and exit codes were excluded from the analysis.

The survey included 134 students in biomedical high schools (G1) and 96 in non-biomedical high schools (G2), 104 in scientific university courses (G3), and 102 in non-scientific university courses (G4).

Among university students, the majority were female, with higher rate (88.2%) among the students of the G4 with respect to those of the G3 (72.1%) (*p* = 0.006).

A range between 71.2% (G3) and 88.8% (G1) of students was Catholic, about 40% of teenagers were engaged, while the rate of university students who were engaged varied between 57.8% (G4) and 60.6% (G3).

All high school students, except one, lived in the family, and only a very few of them had a job (G1:8.2% and G2:7.3%). Among university students, the percentage of those who did not live in the family was greater, with a maximum rate in G4 (24.5%). Among university students, 11.5% in G3 and 13.7% in G4 lived with friends.

Similarly, the number of university students who were employed was higher than those of high schools (Table 1).

Regarding the socio-cultural characteristics of the parents, most of them had a middle school certificate or a Technical–Professional Institute diploma in all groups.

Among the fathers of high school students, about a third were clerks (G1: 30.6% and G2: 31.3%), while 28.4% in G1 and 26.0% in G2 were working men. In G3 and G4 groups, the fathers were mainly clerks (G3: 33.7%; G4: 18.6%), freelance (G3: 27.9%; G4: 29.6%), and working men (G3: 21.2%; G4: 22.5%). Most mothers were housewives with a rate ranging from 38.1% in G1 to 52.0% in G4 (Table 2).

### 3.2. Information Needs about HIV and STDs

More than 60% of students believed in a good or excellent received information on these topics, and this rate increased considerably (*p* = 0.000) after the educational intervention in all groups.

More than 94% of students of the G1 and G2 groups, and 100% of G3 and G4, found it useful to promote information on these issues. Receiving this information created a perception of “awareness” and “safety” in more than 80% and 65% of high school students, as well as in more than 91% and 60% of university students. The percentage of students who perceive these feelings tended to increase further after attending the educational event.

They would have liked to receive information on these topics mainly from sources such as doctors and experts/health professionals. Teachers were also considered to be a reliable source of information on HIV and STDs, mainly by university students (Table 3).

### 3.3. Knowledge on HIV and STDs

The average percentage of correct answers, considering all items together before the educational event, was higher among biomedical high school students (G1: 39.2%) than among non-biomedical high school students (G2: 28.3%). Similarly, the mean rate of correct answers among university students of scientific faculties was 49.5% compared to 35.3% of non-scientific faculties at T0. The highest level of knowledge following the intervention was reached among the students with a biomedical and scientific background with percentages of correct answers corresponding to 65.9% in G1 and 71.7% in G3, respectively (Data not shown).

The highest percentage of correct answers in all T0 groups was related to questions about the transmission of HIV and STDs. In particular, the questions about the spreading modality of STDs (Q8: 78.3% of the correct questions in all groups), the body fluids that can transmit HIV (Q9: 64.5%), and the possibility that a seropositive person may have contracted HIV during a single unprotected sexual intercourse (Q13: 57.2%) were those with a higher frequency of correct responses.

The lowest rates of correct responses at T0 were achieved on the following topics: the definition of HIV-positive person (Q7: 7.8% average percentage of all groups), when to take the HIV-test if you had a risk exposure (Q14: 9.1%), and the availability of a vaccine to prevent chlamydia infection (Q25: 9.8%). The lowest value was found among the students of the G2. Even the definition of “window period” for HIV testing was known by a few students (Q15: 19.0%).

An improvement in knowledge was observed in all groups for all questions. A greater knowledge at T0 was found among students with a scientific background; by comparing these two groups, university students in biotechnology answered more correctly than high school students on 13 out of 15 questions (Table 4).

When evaluating the results of the logistic model for knowledge score between groups, a significant *p*-value occurred between G1 and G2 for the questions Q8, Q11, Q12, and Q16. Instead, a significant difference in the improvement of knowledge between G3 and G4 was observed for the questions Q7, Q10, Q14, and Q16. All other questions showed no significant difference (Table 5).

### 3.4. Risk Perception about HIV and STDs

As for the risk perception of contracting HIV or an STD, a high percentage of students (about 60%) identified it as highly risky (“a lot”) and the concern remained high, even after the educational intervention.

The STDs were considered “a lot” of risk by a high percentage of students, particularly among those of the G3 (94.2%), where only a very small percentage considered these diseases “not at all” dangerous.

More than half of high school students believed that attending someone with STDs as a friend was a great risk, and educational intervention has significantly reduced this belief among G1 (*p* < 0.000) and G2 (*p* < 0.000). On the contrary, a high percentage of university students believed that this behavior was “not at all” risky, and this rate increased further after the intervention.

Almost all the students believe that the use of a condom during sexual intercourse was “widely” safe to avoid infection.

Among high school students, to cause a pregnancy was considered the riskiest consequence of unprotected sex, while among older students, the concern to contract HIV predominated. The latter concern increased in all groups as a result of the educational event.

Most of the students in the four groups believed that the sexual life of their peers was “moderately” safe (range: 84.3–93.2%), and this perception did not change after the intervention. Only a percentage between 1% and 3.1% of students in all groups considered it “widely” safe, both before and after the educational intervention (Table 6).

## 4. Discussion

STDs affect millions of people every year, and their prevention and early detection remain a challenge among the major public health systems. First, the high prevalence of these diseases seems to be associated with a lack of knowledge on the transmission of these infections and on prophylaxis and therapy [38]. Second, young people represent the most exposed category [6]. Therefore, schools and universities are a valuable setting for the huge dissemination of knowledge about HIV and STDs, aimed to reduce at risk behaviors in adolescents and in young adults [39,40]. The main objective of our investigation was to determine—before and after an educational event—the information needs, the level of knowledge, and the risk perception about HIV and STDs of contracting these diseases, among high school and university students attending scientific and non-scientific courses.

Based on study results, students largely believed in their high level of knowledge of HIV and STDs. This perception persisted even after the educational event. In line with the study findings, other investigations showed that most participants rated their knowledge of HIV and STDs as good, although this perception did not reflect their current knowledge [6,41].

The majority of the students recognized the value of promoting information on HIV and STDs and they believed that receiving appropriate information could create a common sense of awareness and safety. They also believed that the most suitable sources from which to receive information about HIV and STDs were physicians, experts, and health professionals. Similarly, Visalli et al. showed that the majority of the considered students attending high schools for humanities and scientific universities complained about the need to receive information from qualified personnel, e.g., doctors and/or health personnel [18].

Moreover, one of the most interesting findings was that parents or relatives were not recognized as reliable sources of information on HIV and STDs. This was consistent with the results of previous studies, in which relatives were not considered a common source of information on sexual matter [18,42]. In fact, talking with the parents about sex was still viewed as unbecoming. In contrast with this common prejudice and reluctance, a good parent–child communication was associated with lower incidence of unwanted pregnancies and STDs in adolescents, as well as with high use of condoms [43,44].

The low initial level of knowledge about HIV and STDs in non-scientific high school and university students addressed in this investigation, confirmed the findings of a similar recent study [18].

In all groups, a significant improvement in knowledge was observed at T1, as reported also in a recent meta-analysis assessing the impact of HIV and other STDs prevention school programmes on adolescents [45].

The percentage of correct answers at the end of the training event among the university students of biotechnology, reaching the highest level, just exceeded 70%. The greatest increase in knowledge in the largest number of responses was found among high school students. This result reflected a greater interest of younger students than their older colleagues. It could have as practical implication the need to plan analogous educational interventions in the high school curricula on these important issues.

Consistently with a previous Italian study, we identified a series of topics in which most students show particular shortfalls. They do not know the term “seropositive”, the difference between HIV and AIDS, the risk of infection during oral sex, the definition of “window period”, and when to take the HIV test after a risky exposure [46].

Furthermore, very few students were able to distinguish which STDs were preventable by vaccination and the role of HPV in the development of cancers in men and women. The belief that the contraceptive pill can serve to prevent the transmission of STDs seemed true in nearly half of the students before the event. Although knowledge improved significantly after the education intervention, our study highlighted a crucial need to gain extensive information on the available prevention tools. Coherently, low levels of vaccination coverage were found in our Local Health Unit even for HPV prevention for which a free and recommended vaccine was available [47].

This lacking knowledge was not surprising, if we think that information on many of these topics was fulfilled through communication campaigns with “short television commercials” or “web pills”, rather than with a health education program as part of the school curriculum.

The high perception of the risk of contracting HIV and STDs in our cohort of students was in contrast to the results of other studies, where a low level of perceived risk was found [26,28].

In an Ethiopian study, the 95.3% of students considered themselves at low risk for contracting HIV [26], while Nyasulu et al. found a risk perception of 24% of getting an STD and 26% for HIV among high school students [28]. However, these were the results obtained in a different setting—such as Africa—so they may be the consequence of a peculiar cultural and religious context.

The younger students downright consider it “widely” risky to attend someone with STDs as a friend. This misperception could be the consequence of the fact that some STDs were almost unknown, or that the methods of prevention and the possible consequences were unfamiliar.

Almost all students were aware that the use of condom reduced the risk of transmission of STDs, even though the greatest concern after unprotected intercourse was the fear of getting pregnant. Other studies reported that young adults used condoms primarily for the prevention of pregnancy and not for avoiding HIV and STDs [10,46,48]. A low perceived risk, often correlated to a low knowledge, can also determine a poor use of the condom to prevent these diseases [49,50]. Even in case the risk of contracting STDs was perceived as low, the concern of an unwanted pregnancy remains high. However, the risk perception of contracting HIV seemed to increase significantly among our students who received training. No significant difference was shown in other studies on the perceived risk of contracting HIV between the intervention group that followed an educational program and the control group [45,51].

Most teens perceived their peers’ sexual life as moderately safe, and this perception did not change even after the educational event. This result seemed to be almost in line with the high perception of their own risk of contracting HIV and STDs that the students expressed in the previous question. Based on these findings, there seemed to be a consistency between the perception of risk deriving from one’s own behaviors and that deriving from the behaviors of one’s peers. Other studies, however, have shown that individuals generally perceived themselves to be at lower risk than others, particularly when the risk was controllable, and this phenomenon was called “optimistic bias” [52,53]. These results constitute a stimulating contribution, due to the fact that it was claimed that peers influenced adolescents’ individual attitudes and behaviors. This peer-to-peer influence not only affected risky behaviors, but also positive protective behaviors, such as adopting contraceptive habits and using condoms [54].

Globally, the risk perception did not change after the educational intervention in almost all target groups. This can be considered as a strengthening theoretical contribution to what literature classically shows regarding the fact that risk perception is influenced by many factors besides knowledge. The psychometric paradigm, in fact, demonstrated that risk perception was constituted by two dimensions, the cognitive and the emotional one [55]. It also assumed that risk was individually perceived based on different psycho-social, cultural, and experiential aspects [56,57]. Another significant consideration was that an adequate perception of risk can represent an important factor to modify and/or to avoid risky sexual behaviors. Nevertheless, future research developments should focus on understanding how to identify and how to facilitate an “adequate” risk perception of STDs.

Some limitations, however, should be considered when analyzing the results of our study. Firstly, it focused mainly on the assessment of knowledge about HIV, while only a few questions about the other STDs were included in the analysis. Moreover, we used just few measurement elements to assess the risk perception of HIV and STDs, although the importance of multiple items in risk perception analysis has been demonstrated [58]

Another limitation of our research was that the effect of the intervention on knowledge and other constructs was evaluated immediately after the intervention. This circumstance may not have allowed students to use time to properly assimilate and process the provided knowledge. In fact, studies on the effectiveness of health educational programs underlined how interventions can influence sex-related behaviors through cognitive processes, requiring a certain period of time [1].

## 5. Conclusions

The study results showed that knowledge about some aspects of HIV and other STDs was inadequate among both scientific and non-scientific high school and university students. Furthermore, it highlighted the effectiveness of an educational intervention to improve the limited baseline knowledge of HIV and STDs, particularly among students attending non-biomedical high schools and university faculties. Another important evidence was that each target group revealed certain specific topics that were inadequate and needed specific and continuous learning programs.

These results, based also on the information need expressed by students and the concern about these infections, supported the necessity to adopt educational programs aimed to provide information on these issues for the adolescents and young adults. They finally reinforced the need to encourage health promotion campaigns addressing messages targeted on each different target group.

## Figures and Tables

**Table 1:** Socio-demographic characteristics of students.

Variables	Group 1 (*n* = 134) *n* (%)	Group 2 (*n* = 96) *n* (%)	*p*-Value	Group 3 (*n* = 104) *n* (%)	Group 4 (*n* = 102) *n* (%)	*p*-Value	*p*-Value
**Gender**							
Male, *n* (%)	51 (38.1)	49 (51.0)		29 (27.9)	12 (11.8)		
Female, *n* (%)	83 (61.9)	47 (49.0)	0.068 °	75 (72.1)	90 (88.2)	0.006 °	<0.000 °
**Age** (mean ± SD)	16.6 ± 1.0	16.7 ± 0.9	0.407 ^	20.6 ± 1.1	22.3 ± 3.3	<0.000 ^	<0.001 ^#^
**Religion**							
Catholic, *n* (%)	119 (88.8)	83 (86.5)		74 (71.2)	85 (83.3)		
Islamic, *n* (%)	2 (1.5)	1 (1.0)		- (-)	- (-)		
Orthodox, *n* (%)	1 (0.7)	1 (1.0)		- (-)	- (-)		
Not religious, *n* (%)	10 (7.5)	9 (9.4)		29 (27.9)	16 (15.7)		-
Other, *n* (%)	2 (1.5)	2 (2.1)	0.959 *	1 (1.0)	1 (1.0)	0.059 *	
**Relationship status**							
Single, *n* (%)	75 (56.0)	57 (59.4)		41 (39.4)	43 (42.2)		
Engaged, *n* (%)	59 (44.0)	39 (40.6)	0.704 °	63 (60.6)	59 (57.8)	0.797 °	0.006 °
**Household status**							
In family, *n* (%)	134 (100)	95 (99.0)		90 (86.5)	77 (75.5)		
With relatives, *n* (%)	- (-)	1 (1.0)		1 (1.0)	- (-)		
Alone, *n* (%)	- (-)	- (-)		- (-)	6 (5.9)		
With friends, *n* (%)	- (-)	- (-)		12 (11.5)	14 (13.7)		
Other, *n* (%)	- (-)	- (-)	0.417 *	1 (1.0)	5 (4.9)	0.014 *	-
**Employment**							
No, *n* (%)	123 (91.8)	89 (92.7)		91 (87.5)	76 (74.5)		
Yes, *n* (%)	11 (8.2)	7 (7.3)	0.995°	13 (12.5)	26 (25.5)	0.028 °	0.000 °

° Chi Square test; * Fisher’s Exact Test; ^ t test Two-tailed; # ANOVA; - 0 value.

**Table 2:** Socio-cultural characteristics of parents.

Variables	Group 1 (*n* = 134) *n* (%)	Group 2 (*n* = 96) *n* (%)	*p*-Value	Group 3 (*n* = 104) *n* (%)	Group 4 (*n* = 102) *n* (%)	*p*-Value
**Education level**						
**Father**						
Elementary school, *n* (%)	12 (9.0)	4 (4.2)		1 (1.0)	12 (11.8)	
Middle school, *n* (%)	60 (44.8)	45 (46.9)		31 (29.8)	47 (46.1)	
High school, *n* (%)	7 (5.2)	7 (7.3)		11 (10.6)	9 (22.5)	
Technical-professional school, *n* (%)	39 (29.1)	30 (31.3)		43 (41.3)	23 (22.5)	
Bachelor’s or Master’s degree, *n* (%)	10 (7.5)	7 (7.3)		11 (10.6)	9 (8.8)	
Advanced Professional degree, *n* (%)	5 (3.7)	2 (2.1)		6 (5.8)	2 (2.0)	
Not answered	1 (0.7)	1 (1.0)	0.817	1 (1.0)	- (-)	0.001
**Mother**						
Elementary school, *n* (%)	4 (3.0)	1 (1.0)		2 (1.9)	14 (13.7)	
Middle school, *n* (%)	53 (39.6)	42 (43.8)		33 (31.7)	41 (40.2)	
High school, *n* (%)	18 (13.4)	10 (10.4)		15 (14.4)	18 (17.6)	
Technical-professional school, *n* (%)	43 (32.1)	33 (34.4)		32 (30.8)	21 (20.6)	
Bachelor’s or Master’s degree, *n* (%)	10 (7.5)	7 (7.3)		16 (15.4)	8 (7.8)	
Advanced Professional degree, *n* (%)	5 (3.7)	3 (3.1)		5 (4.8)	- (-)	
Not answered	1 (0.7)	- (-)	0.869	- (-)	- (-)	0.001
**Occupation**						
**Father**						
Entrepreneur, *n* (%)	12 (9.0)	14 (14.6)		5 (4.8)	12 (11.8)	
Freelance, *n* (%)	26 (19.4)	16 (16.7)		29 (27.9)	20 (29.6)	
Manager, *n* (%)	3 (2.2)	1 (1.0)		2 (1.9)	2 (2.0)	
Clerk, *n* (%)	41 (30.6)	30 (31.3)		35 (33.7)	19 (18.6)	
Teacher, *n* (%)	3 (2.2	- (-)		5 (4.8)	2 (2.0)	
Working men, *n* (%)	38 (28.4)	25 (26.0)		22 (21.2)	23 (22.5)	
Home worker, *n* (%)	- (-)	- (-)		- (-)	- (-)	
Unemployed, *n* (%)	4 (3.0)	6 (6.3)		2 (1.9)	4 (3.9)	
Other, *n* (%)	4 (3.0)	2 (2.1)		4 (3.8)	20 (19.6)	
Not answered	3 (2.2)	2 (2.1)	0.532	- (-)	- (-)	0.003
**Mother**						
Entrepreneur, *n* (%)	5 (3.7)	6 (6.3)		1 (1.0)	1 (1.0)	
Freelance, *n* (%)	7 (5.2)	6 (6.3)		11 (10.6)	5 (4.9)	
Manager, *n* (%)	3 (2.2)	- (-)		- (-)	- (-)	
Clerk, *n* (%)	44 (32.8)	20 (20.8)		25 (24.0)	17 (16.7)	
Teacher, *n* (%)	3 (2.2)	8 (8.3)		8 (7.7)	4 (3.9)	
Working women, *n* (%)	9 (6.7)	5 (5.2)		5 (4.8)	10 (9.8)	
Home worker, *n* (%)	1 (0.7)	6 (6.3)		0 (0)	3 (2.9)	
Housewife, *n* (%)	51 (38.1)	40 (41.7)		43 (41.3)	53 (52.0)	
Unemployed, *n* (%)	9 (6.7)	4 (4.2)		7 (6.7)	3 (2.9)	
Other, *n* (%)	1 (0.7)	1 (1.0)		2 (1.9)	5 (4.9)	
Not answered	1 (0.7)	- (-)	0.051	2 (1.9)	1 (1.0)	0.133

Fisher’s Exact Test; - 0 value.

**Table 3:** Information needs about HIV and STDs before (T0) and after (T1) the educational intervention.

Item	Group 1 (*n* = 134) % T0 T1		*p*-Value	Group 2 (*n* = 96) % T0 T1		*p*-Value	Group 3 (*n* = 104) % T0 T1		*p*-Value	Group 4 (*n* = 102) % T0 T1		*p*-Value
Q1. How do you evaluate information you acquired about HIV and STDs? *												
Absent	10 (7.5)	1 (0.8)		3 (3.2)	1 (1.0)		2 (1.9)	- (-)		3 (2.9)	-	
Scarce	33 (24.6)	2 (1.6)		15 (15.6)	2 (2.1)		29 (27.9)	1 (1.0)		28 (27.5)	1 (1.0)	
Good	63 (47.0)	42 (31.3)		47 (49.0)	53 (55.2)		54 (51.9)	47 (45.2)		48 (47.1)	44 (43.1)	
Excellent	24 (17.9)	87 (64.9)		17 (17.7)	38 (39.6)		8 (7.7)	56 (53.8)		13 (12.7	57 (55.9)	
I don’t know	2 (1.5)	1 (0.7)		13 (13.5)	2 (2.1)		11 (10.6)	- (-)		9 (8.8)	- (-)	
Not answered	2 (1.5)	1 (0.7)	0.000	1 (1.0)	- (-)	0.000	-	- (-)	0.000	1 (1.0)	- (-)	0.000
**Q2. Do you think that promoting information about HIV and STDs is: ^**												
Useful	126 (94.0)	131 (97.8)		93 (96.9)	90 (93.8)		104 (100)	102 (98.1)		102 (100)	100 (98.0)	
Useless	2 (1.5)	1 (0.7)		- (-)	4 (4.2)		- (-)	2 (1.9)		- (-)	1 (1.0)	
I don’t know	4 (3.0)	2 (1.5)		3 (3.1)	1 (1.0)		- (-)	- (-)		- (-)	- (-)	
Not answered	2 (1.5)	-	NA	- (-)	1 (1.0)	NA	- (-)	- (-)	NA	- (-)	1 (1.0)	NA
**Q3. Do you think that receiving information about HIV and STD create °: ^**												
Awareness	108 (80.6)	122 (91.0)	0.008	79 (82.3)	82 (85.4)	0.719	101 (97.1)	102 (98.1)	1.000	93 (91.2)	98 (96.1)	0.267
Safety	92 (68.71)	93 (69.4)	1.000	63 (65.6)	62 (64.6)	1.000	63 (60.6)	74 (71.2)	0.029	65 (63.7)	81 (79.4)	0.018
Anxiety	22 (16.4)	19 (14.2)	0.646	12 (12.5)	22 (22.9)	0.112	12 (11.5)	6 (5.8)	0.041	7 (6.9)	4 (3.9)	0.546
Discomfort	9 (6.7)	6 (4.5)	0.371	3 (3.1)	5 (5.2)	0.724	- (-)	- (-)	NA	- (-)	- (-)	NA
Confusion	3 (2.2)	2 (1.5)	1.000	1 (1.0)	4 (4.2)	0.371	- (-)	1 (1.0)	NA	- (-)	- (-)	NA
Nothing	- (-)	- (-)	NA	2 (2.1)	1 (1.0)	1.000	- (-)	- (-)	NA	- (-)	- (-)	NA
Other	1 (0.7)	1 (0.7)		- (-)	1 (1.0)		- (-)	- (-)		1 (1.0)	- (-)	
Not Answered	- (-)	- (-)		- (-)	- (-)		- (-)	- (-)		- (-)	- (-)	
**Q4. Which of the following sources do you consider as reliable to get information about HIV and STDs °: ^**												
Parents/relatives	7 (5.2)	19 (14.2)	0.003	25 (26.0)	27 (28.1)	0.814	17 (16.3)	23 (22.1)	0.149	15 (14.7)	21 (20.6)	0.327
Physicians	58 (43.3)	98 (73.1)	0.000	65 (67.7)	73 (76.0)	0.099	81 (77.9)	90 (86.5)	0.052	83 (81.4)	83 (81.49)	1.000
Teacher	5 (3.7)	32 (23.9)	0.000	25 (26.0)	24 (25.0)	1.000	56 (53.8)	64 (61.5)	0.186	54 (52.9)	40 (39.2)	0.066
Radio	1 (0.7)	2 (1.5)	1.000	- (-)	2 (2.1)	NA	10 (9.6)	12 (11.5)	0.683	3 (2.9)	4 (3.9)	1.000
Experts/Health professionals	49 (36.6)	97 (72.4)	0.000	60 (62.5)	61 (63.5)	1.000	64 (61.5)	70 (67.3)	0.327	69 (67.6)	81 (79.4)	0.090
	- (-)	7 (5.2)	NA	6 (6.3)	6 (6.3)	1.000	18 (17.3)	16 (15.4)	0.752	12 (11.8)	13 (12.7)	1.000
Encyclopaedias	3 (2.2)	3 (2.2)	1.000	15 (15.6)	9 (9.4)	0.041	5 (4.8)	6 (5.8)	1.000	3 (2.9)	3 (2.9)	1.000
Friends	5 (3.7)	20 (14.9)	0.002	17 (17.7)	17 (17.7)	1.000	29 (27.9)	37 (35.6)	0.136	15 (14.7)	19 (18.6)	0.596
Internet	2 (1.5)	18 (13.4)	0.000	10 (10.4)	13 (13.5)	0.505	36 (34.6)	41 (39.4)	0.302	22 (21.6)	29 (28.4)	0.360
TV												
Magazines/newspapers	1 (0.7)	2 (1.5)	1.000	3 (3.1)	4 (4.2)	1.000	7 (6.7)	9 (8.7)	0.683	8 (7.8)	10 (9.8)	0.814
for adults												
Magazines for teenagers/	3 (2.2)	15 (11.2)	0.004	8 (8.3)	8 (8.3)	1.000	17 (16.3)	16 (15.4)	1.000	15 (14.7)	15 (14.7)	1.000
young people												
Other	- (-)	- (-)		- (-)	- (-)		1 (1.0)	- (-)		- (-)	1 (1.0)	
Not Answered	- (-)	- (-)		- (-)	- (-)		-	- (-)		- (-)	1 (1.0)	

° Possibility of multiple answer; NA: Not applicable; * Wilcoxon signed-rank test; ^ McNemar test; - 0 value.

**Table 4:** Knowledge about HIV and STDs before (T0) and after (T1) the educational intervention.

Item	Group 1 (*n* = 134) % T0 T1		*p*-Value	Group 2 (*n* = 96) % T0 T1		*p*-Value	Group 3 (*n* = 104) % T0 T1		*p*-Value	Group 4 (*n* = 102) % T0 T1		*p*-Value
**Q5. Which of the following diseases is not a STD?**												
	59 (44.0)	103 (76.9)	0.000	12 (12.5)	52 (54.2)	0.000	53 (51.0)	93 (89.4)	0.000	41 (40.2)	81 (79.4)	0.000
**Q6. Is there any difference between HIV and AIDS?**												
	69 (51.5)	99 (73.9)	0.000	35 (36.5)	63 (65.6)	0.000	60 (57.7)	66 (63.5)	0.149	33 (32.4)	50 (49.0)	0.022
**Q7. Who is a HIV-positive?**												
	11 (8.2)	23 (17.2)	0.045	4 (4.2)	21 (21.9)	0.000	14 (13.5)	53 (51.0)	0.000	6 (5.9)	29 (28.4)	0.000
**Q8. Which of the following is not a spreading modality for STDs?**												
	103 (76.9)	122 (91.0)	0.002	64 (66.7)	73 (76.0)	0.095	96 (92.3)	99 (95.2)	0.371	79 (77.5)	89 (87.3)	0.089
**Q9. Which body fluids can transmit HIV?**												
	99 (73.9)	125 (93.3)	0.000	50 (52.1)	81 (84.4)	0.000	79 (76.0)	89 (85.6)	0.066	57 (55.9)	75 (73.5)	0.012
**Q10. Which of the following activities can transmit HIV/AIDS?**												
	49 (36.6)	69 (51.5)	0.001	12 (12.5)	38 (39.6)	0.000	37 (35.6)	71 (68.3)	0.000	29 (28.4)	45 (44.1)	0.030
**Q11. Can HIV be transmitted through close contacts or kisses?**												
	59 (44.0)	123 (91.8)	0.000	37 (38.5)	76 (79.2)	0.000	70 (67.3)	77 (74.0)	0.248	48 (47.1)	74 (72.5)	0.001
**Q12. Can a person contract HIV through oral sexual intercourse?**												
	33 (24.6)	83 (61.9)	0.000	25 (26.0)	47 (49.0)	0.002	37 (35.6)	48 (46.2)	0.015	25 (24.5)	40 (39.2)	0.029
**Q13. A seropositive person can get HIV if he/she has sexual intercourses with another person?**												
	87 (64.9)	119 (88.8)	0.000	55 (57.3)	77 (80.2)	0.000	57 (54.8)	60 (57.7)	0.450	53 (52.0)	57 (55.9)	0.626
**Q14. When do you have to take the HIV test if you had an unprotected sexual intercourse?**												
	6 (4.5)	49 (36.6)	0.000	-	29 (30.2)	0.000	25 (24.0)	57 (54.8)	0.000	8 (7.8)	33 (32.4)	0.000
**Q15. What is the window period for HIV testing?**												
	18 (13.4)	83 (61.9)	0.000	10 (10.4)	49 (51.0)	0.000	33 (31.7)	85 (81.7)	0.000	21 (20.6)	78 (76.5)	0.000
**Q16. Can oral contraceptive pill prevent the transmission of sexual infections?**												
	75 (56.0)	105 (78.4)	0.000	43 (44.8)	58 (60.4)	0.012	83 (79.8)	88 (84.6)	0.441	58 (56.9)	69 (67.6)	0.114
**Q17. Is there a vaccine to prevent chlamydia infection?**												
	13 (9.7)	49 (36.6)	0.000	4 (4.2)	28 (29.2)	0.000	21 (20.2)	66 (63.5)	0.000	5 (4.9)	61 (59.8)	0.000
**Q18. Can a patient affected by AIDS heal?**												
	55 (41.0)	83 (61.9)	0.000	34 (35.4)	64 (66.7)	0.000	53 (51.0)	75 (72.1)	0.000	39 (38.2)	60 (58.8)	0.003
**Q19. Can HPV lead to cancer in men and women?**												
	52 (38.8)	90 (67.2)	0.000	23 (24.0)	54 (56.3)	0.000	54 (51.9)	91 (87.5)	0.000	38 (37.3)	90 (88.2)	0.000

McNemar test.

**Table 5:** Comparison of knowledge scores after educational intervention between G1 and G2; G3 and G4.

Items	OR (95% CI)	*p*-Value	OR (95% CI)	*p*-Value
**Q5.**	0.57 (0.31–1.05)	0.071	0,48 (0.22–1.07)	0.073
**Q6.**	0.81 (0.44–1.47)	0.480	0.82 (0.44–1.54)	0.542
**Q7.**	1.39 (0.71–2.69)	0.336	0.41 (0.23–0.73)	0.002
**Q8.**	0.34 (0.16–0.76)	0.008	0.47 (0.15–1.42)	0.179
**Q9.**	0.45(0.18–1.11)	0.083	0.54 (0.26–1.11)	0.093
**Q10.**	0.98 (0.54–1.78)	0.959	0.37 (0.21–0.67)	0.001
**Q11.**	0.35 (0.16–0.77)	0.009	1.09 (0.57–2.06)	0.798
**Q12.**	0.58 (0.34–0.99)	0.048	0.91 (0.49–1.67)	0.753
**Q13.**	0.55 (0.25–1.21)	0.140	0.99 (0.51–1.92)	0.968
**Q14.**	0.77 (0.44–1.36)	0.369	0.43 (0.24–0.77)	0.004
**Q15.**	0.65 (0.38–1.11)	0.114	0.76 (0.39–1.51)	0.439
**Q16.**	0.45 (0.23–0.86)	0.015	0.41 (0.20–0.82)	0.011
**Q17.**	0.75 (0.42–1.32)	0.317	0.96 (0.54–1.71)	0.881
**Q18.**	1.31 (0.74–2.30)	0.350	0.62 (0.34–1.13)	0.119
**Q19.**	0.70 (0.40–1.22)	0.207	1.14 (0.49–2.66)	0.762

Baseline adjusted logistic model.

**Table 6:** Risk perception about HIV and STDs, before (T0) and after (T1) the educational intervention.

Item	Group 1 (*n* = 134) % T0 T1		*p*-Value	Group 2 (*n* = 96) % T0 T1		*p*-Value	Group 3 (*n* = 104) % T0 T1		*p*-Value	Group 4 (*n* = 102) % T0 T1		*p*-Value
**Q20. How much do you perceive being at risk of contracting HIV or an STD?**												
A lot	85 (63.4)	78 (58.2)		60 (62.5)	64 (66.7)		52 (50.0)	54 (51.9)		68 (66.8)	67 (65.7)	
Moderately	35 (26.2)	39 (29.1)		29 (30.2)	18 (18.7)		29 (27.9)	36 (34.6)		26 (25.4)	28 (27.4)	
Not at all	14 (10.4)	17 (12.7)		7 (7.3)	14 (14.6)		23 (22.1)	13 (12.5)		8 (7.8)	7 (6.9)	
Not answered	- (-)	- (-)	0.173 °	- (-)	- (-)	0.651 °	- (-)	1 (1.0)	0.027 °	- (-)	- (-)	0.916 °
**Q21. Taking into account that they can be cured, how dangerous are STDs?**												
A lot	104 (77.7)	105 (78.4)		70 (72.9)	74 (77.1)		98 (94.2)	98 (94.2)		83 (81.4)	88 (86.3)	
Moderately	14 (10.4)	3 (2.2)		16 (16.7)	7 (7.3)		3 (2.9)	1 (1.0)		10 (9.8)	3 (2.9)	
Not at all	15 (11.2)	26 (19.4)		10 (10.4)	15 (15.6)		3 (2.9)	4 (3.8)		9 (8.8)	11 (10.8)	
Not answered	1 (0.7)	- (-)	0.208 °	- (-)	- (-)	0.757 °	- (-)	1 (1.0)	1.000 °	- (-)	- (-)	0.983 °
**Q22. How risky is friendly frequenting a person with an STD?**												
A lot	75 (56.0)	50 (37.3)		57 (59.4)	44 (45.8)		24 (23.1)	28 (26.9)		35 (34.3)	44 (43.1)	
Moderately	12 (9.0)	5 (3.7)		18 (18.8)	6 (6.3)		8 (7.7)	2 (1.9)		16 (15.7)	2 (2.0)	
Not at all	47 (35.0)	79 (59.0)		20 (20.8)	46 (47.9)		72 (69.2)	73 (70.2)		51 (50.0)	56 (54.9)	
Not answered	- (-)	- (-)	0.955 °	1 (1.0)		0.000 °	- (-)	1 (1.0)	0.813 °	- (-)	- (-)	0.746 °
**Q23. How safe is using condom during sexual intercourse to avoid an STDs?**												
A lot	127 (94.8)	131 (97.9)		83 (86.5)	90 (93.8)		103 (99.0)	101 (97.1)		100 (98.0)	100 (98.0)	
Moderately	2 (1.5)	1 (0.7)		10 (10.4)	4 (4.2)		-	-		2 (2.0)	2 (2.0)	
Not at all	5 (3.7)	1 (0.7)		2 (2.1)	1 (1.0)		1 (1.0)	2 (1.9)		- (-)	- (-)	
Not answered	- (-)	1 (0.7)	0.083 °	1 (1.0)	1 (1.0)	0.065 °	- (-)	1 (1.0)	0.773 °	- (-)	- (-)	1.000 °
**Q24. Which is the most risky consequence of unprotected sex?**												
Contracting HIV	40 (29.9)	57 (42.5)		30 (31.3)	48 (50.0)		55 (52.8)	70 (67.3)		45 (44.1)	56 (54.9)	
Causing a pregnancy	63 (47.0)	41 (30.6)		45 (46.9)	23 (24.0)		27 (26.0)	13 (12.5)		30 (29.4)	20 (19.6)	
Contracting another STD	22 (16.4)	25 (18.7)		17 (17.7)	10 (10.4)		16 (15.4)	16 (15.4)		21 (20.6)	24 (23.5)	
Other	9 (6.7)	10 (7.5)		4 (4.2)	14 (14.6)		6 (5.8)	4 (3.8)		6 (5.9)	2 (2.0)	
Not answered	- (-)	1 (0.7)	NA *	- (-)	1 (1.0)	0.000 *	- (-)	1 (1.0)	NA	- (-)	- (-)	0.163 *
**Q25. How safe do you think your peers’ sexual life is?**												
A lot	3 (2.2)	2 (1.5)		2 (2.1)	3 (3.1)		2 (1.9)	1 (1.0)		-	-	
Moderately	115 (85.9)	113 (84.3)		85 (88.5)	86 (89.6)		96 (92.3)	97 (93.2)		93 (91.2)	93 (91.2)	
Not at all	16 (11.9)	19 (14.2)		9 (9.4)	6 (6.3)		5 (4.8)	5 (4.8)		9 (8.8)	9 (8.8)	
Not answered	- (-)	- (-)	0.359 °	- (-)	1 (1.0)	0.267 °	1 (1.0)	1 (1.0)	0.777 °	- (-)	- (-)	1.000 °

NA: Not applicable; ° Wilcoxon signed-rank test; * McNemar Test; - 0 value.

## Data Availability

The data presented in this study are available on request from the corresponding author. The data are not publicly available due to privacy.

## Supplementary Materials

The following are available online at https://www.mdpi.com/1660-4601/18/4/2069/s1: Questionnaire on knowledge, information needs, and risk perception about HIV and Sexually Transmitted Infections on high school and university students.
