# Supplementary material for: Knowledge, Information Needs and Risk Perception about HIV and Sexually Transmitted Diseases after an Education Intervention on Italian High School and University Students
Source: Int J Environ Res Public Health. 2021 Feb 20;18(4):2069. doi: 10.3390/ijerph18042069 (PMC7923805; doi:10.3390/ijerph18042069)
Supplement: Supplementary file 1 [file ijerph-18-02069-s001.pdf]

Questionnaire on knowledge, information needs and risk perception about HIV and Sexually Transmitted Infections on high school and university students

ID number _____

The purpose of this questionnaire is to survey knowledge, information needs and risk perception about HIV and STIs of high school and university students. This is an anonymous questionnaire only for research use.

Note: 1. Please tick “X” in “☐” the appropriate option.
2. All options are single (when more than one answer can be crossed, it is specifically indicated in the question).

Section A. Socio-demographic and cultural characteristics

Date of birth (YYYY/MM/DD): ______________________________

Gender:

☐ Male
☐ Female

Religion:

☐ Catholic
☐ Islamic
☐ Orthodox
☐ Not religious
☐ Other

Relationship status:

☐ Single
☐ Engaged

Household status:

☐ In family
☐ With relatives
☐ Alone
☐ With friends
☐ Other

Employment:

☐ No
☐ Yes

Education level:

	**Father**	**Mother**
Elementary school	☐	☐
Middle school	☐	☐
High school	☐	☐
Technical-professional school	☐	☐
Bachelor’s or Master’s degree	☐	☐
Advanced Professional degree	☐	☐
Not answered	☐	☐

Occupation father:

Entrepreneur	☐
Freelance	☐
Manager	☐
Clerk	☐
Teacher	☐
Working men	☐
Home worker	☐
Unemployed	☐
Other	☐
Not answered	☐

Occupation mother:

Entrepreneur	☐
Freelance	☐
Manager	☐
Clerk	☐
Teacher	☐
Working women	☐
Home worker	☐
Housewife	☐
Unemployed	☐
Other	☐
Not answered	☐

Section B. Information needs about HIV and STIs

Q1. How do you evaluate information you acquired about HIV and STIs?

☐ Absent
☐ Scarce
☐ Good
☐ Excellent
☐ I don’t know

Q2. Do you think that promoting information about HIV and STDs is:

☐ Useful
☐ Useless
☐ I don’t know

Q3. Do you think that receiving information about HIV and STD create (possibility of multiple answer):

☐ Awareness
☐ Safety
☐ Anxiety
☐ Discomfort
☐ Confusion
☐ Nothing
☐ Other

Q4. Which of the following sources do you consider as reliable to get information about HIV and STIs (possibility of multiple answer):

☐ Parents/relatives
☐ Physicians
☐ Teachers
☐ Radio
☐ Experts/Health professionals
☐ Encyclopedias
☐ Friends
☐ Internet
☐ TV
☐ Magazines/newspapers for adults
☐ Magazines for teenagers/young people
☐ Other

Section C. Knowledge about HIV and STIs

Q5. Which of the following diseases is not a STI?

☐ Syphilis
☐ Hepatitis B
☐ Chlamydia
☐ Papillomavirus
☐ Pertussis
☐ HIV/AIDS
☐ Gonorrhea

Q6. Is there any difference between HIV and AIDS?

☐ Yes, AIDS is the infection which causes HIV disease
☐ No, there is no difference
☐ Yes, AIDS is a syndrome caused by HIV infection
☐ I don’t know

Q7. Who is a HIV-positive?

☐ A person whose test detected antibodies to HIV in the blood
☐ A person whose test found no HIV antibodies in the blood
☐ A patient with AIDS
☐ I don’t know

Q8. Which of the following is not a spreading modality for STIs?

☐ Sharing injection needles
☐ Sharing objects
☐ Sexual intercourse
☐ Blood transfusion
☐ Mother-to-child transmission
☐ I don’t know

Q9. Which body fluids can transmit HIV?

☐ Blood, Semen, Vaginal discharge
☐ Sweat, Saliva, Urine
☐ Blood, Saliva, Semen
☐ I don’t know

Q10. Which of the following activities can transmit HIV/AIDS?

☐ Homo/heterosexual intercourse
☐ Only anal intercourse
☐ Kissing, petting, vaginal intercourse
☐ Insect bites
☐ I don’t know

Q11. Can HIV be transmitted through closeness or kisses?

☐ Yes
☐ No
☐ It depends on the person’s immune system
☐ Yes, only when the disease is at an advanced stage
☐ I don’t know

Q12. Can a person contract HIV through oral sexual intercourse?

☐ Yes
☐ No
☐ Maybe yes, but it was never detected
☐ Yes, mainly in cases where semen or vaginal fluid laps the mucous membranes
☐ I don’t know

Q13. A seropositive person can transmit HIV if he/she has sexual intercourse with another person:

☐ One time
☐ Al least 2 times
☐ >3 times
☐ Many times, but there is no a definite number
☐ I don’t know

Q14. When do you have to take the HIV test if you had an unprotected sexual intercourse?

☐ After the incubation period
☐ The day after
☐ After about 12 weeks
☐ Depends on whether you are a boy or a girl
☐ I don’t know

Q15. What is the window period for HIV testing?

☐ The time between contagion and seroconversion
☐ The time between infection and onset of symptoms
☐ The time between the administration of test and the obtainment of results
☐ I don’t know

Q16. Can the oral contraceptive pill prevent the STIs?

☐ Yes
☐ No
☐ I don’t know

Q17. Is there a vaccine to prevent chlamydia infection?

☐ Yes
☐ No
☐ I don’t know

Q18. Can a patient affected by AIDS heal?

☐ Yes
☐ No
☐ Maybe, with an appropriate therapeutic plan
☐ Yes, if he/she no longer has risky sexual intercourses
☐ I don’t know

Q19. Can HPV lead to cancer in men and women?

☐ Yes
☐ No
☐ I don’t know

Section D. Risk perception about HIV and STIs

Q20. How much do you perceive being at risk of contracting HIV or an STI?

☐ A lot
☐ Moderately
☐ Not at all

Q21. Taking into account that they can be cured, how dangerous are STIs?

☐ A lot
☐ Moderately
☐ Not at all

Q22. How risky is friendly frequenting a person with an STIs?

☐ A lot
☐ Moderately
☐ Not at all

Q23. How safe is using condom during sexual intercourse to avoid an STIs?

☐ A lot
☐ Moderately
☐ Not at all

Q24. Which is the most risky consequence of unprotected sex?

☐ Contracting HIV
☐ Causing a pregnancy
☐ Contracting another STI
☐ Other

Q25. How safe do you think your peers' sexual life is?

☐ A lot
☐ Moderately
☐ Not at all

Date (YYYY/MM/DD): ______________________________

Thank you for participating!!!
